# The spleen-strengthening and liver-draining herbal formula treatment of non-alcoholic fatty liver disease by regulation of intestinal flora in clinical trial

**DOI:** 10.3389/fendo.2022.1107071

**Published:** 2023-01-19

**Authors:** Dengcheng Hui, Lu Liu, Nisma Lena Bahaji Azami, Jingru Song, Yanping Huang, Wan Xu, Chao Wu, Dong Xie, Yulang Jiang, Yanqin Bian, Mingyu Sun

**Affiliations:** ^1^ Shuguang Hospital Affiliated to Shanghai University of Traditional Chinese Medicine, Shanghai, China; ^2^ Shanghai University of Traditional Chinese Medicine, Shanghai, China; ^3^ Institute of Liver Diseases, Key Laboratory of Liver and Kidney Diseases, Shuguang Hospital Affiliated to Shanghai University of Traditional Chinese Medicine, Shanghai, China; ^4^ Department of Gastroenterology, Hangzhou TCM Hospital Affiliated to Zhejiang Chinese Medical University, Hangzhou, Zhejiang, China; ^5^ Department of Good Clinical Practice Office, Tongren Hospital, Shanghai Jiao Tong University School of Medicine, Shanghai, China; ^6^ Arthritis Institute of Guanghua Hospital, Shanghai University of Traditional Chinese Medicine, Shanghai, China

**Keywords:** non-alcoholic fatty liver disease, traditional Chinese medicine, spleen-strengthening and liver-draining formula, intestinal flora, glucolipid metabolism

## Abstract

**Objective:**

As a metabolic disease, one important feature of non-alcoholic fatty liver disease (NAFLD) is the disturbance of the intestinal flora. Spleen-strengthening and liver-draining formula (SLF) is a formula formed according to the theory of “One Qi Circulation” (Qing Dynasty, 1749) of Traditional Chinese Medicine (TCM), which has shown significant therapeutic effect in patients with NAFLD in a preliminary clinical observation. In this study, we aim to explore the mechanism of SLF against NAFLD, especially its effect on glucolipid metabolism, from the perspective of intestinal flora.

**Methods:**

A prospective, randomized, controlled clinical study was designed to observe the efficacy and safety of SLF in the treatment of NAFLD. The study participants were randomly and evenly divided into control group and treatment group (SLF group). The control group made lifestyle adjustments, while the SLF group was treated with SLF on top of the control group. Both groups were participated in the study for 12 consecutive weeks. Furthermore, the feces of the two groups were collected before and after treatment. The intestinal flora of each group and healthy control (HC) were detected utilizing 16S rRNA gene sequencing.

**Results:**

Compared with the control group, the SLF group showed significant improvements in liver function, controlled attenuation parameter (CAP), and liver stiffness measurement (LSM), meanwhile, patients had significantly lower lipid and homeostasis model assessment of insulin resistance (HOMA-IR) with better security. Intestinal flora 16S rRNA gene sequencing results indicated reduced flora diversity and altered species abundance in patients with NAFLD. At the phylum level, *Desulfobacterota* levels were reduced. Although *Firmicutes* and *Bacteroidetes* did not differ significantly between HC and NAFLD, when grouped by alanine transaminase (ALT) and aspartate transaminase (AST) levels in NAFLD, *Firmicutes* levels were significantly higher in patients with ALT or AST abnormalities, while *Bacteroidetes* was significantly lower. Clinical correlation analysis showed that *Firmicutes* positively correlated with gender, age, ALT, AST, LSM, and Fibroscan-AST (FAST) score, while the opposite was true for *Bacteroidetes*. At the genus level, the levels of *Alistipes*, *Bilophila*, *Butyricimonas*, *Coprococcus*, *Lachnospiraceae_NK4A136* group *Phascolarctobacterium*, *Ruminococcus*, *UCG-002*, and *UCG-003* were reduced, whereas abundance of *Tyzzerella* increased. There was no statistically significant difference in *Firmicutes* and *Bacteroidota* levels in the SLF group before and after treatment, but both bacteria tended to retrace. At the genus level, *Coprococcus* (*Lachnospiraceae* family), *Lachnospiraceae_NK4A136 group* (*Lachnospiraceae* family), and *Ruminococcus* (*Ruminococcaceae* family) were significantly higher in the SLF group after treatment, and there was also a tendency for *Bilophila* (*Desulfovibrionaceae* family) to be back-regulated toward HC.

**Conclusions:**

SLF can improve liver function and glucolipid metabolism in patients with NAFLD and lower down liver fat content to some extent. SLF could be carried out by regulating the disturbance of intestinal flora, especially *Coprococcus*, *Lachnospiraceae_NK4A136* group, and *Ruminococcus* genus.

## Introduction

Non-alcoholic fatty liver disease (NAFLD), is common disease characterized by steatosis in more than 5% of hepatocytes with no excessive alcohol consumption, and is a form of liver reaction to a metabolic syndrome (1). The incidence of NAFLD in adults ranges from 20 to 30% (2), which is expected to continue to rise because of the ongoing obesity epidemic that begins in childhood, the increase in diabetes, as well as other factors in recent years (3). Non-alcoholic steatohepatitis (NASH) is the further stage of non-alcoholic fatty liver, accompanied by liver inflammation and hepatocellular ballooning, which may further develop into liver cirrhosis and hepatocellular carcinoma with a high probability (4). There are no specific therapeutic drugs for NAFLD approved by the Food and Drug Administration at present, so the investigation of effective therapeutics is warranted.

In recent years, Traditional Chinese Medicine (TCM) has been favored by a growing number of patients because of its good efficacy with few side effects. There is a promising development and application prospect in the prevention and treatment of NAFLD with TCM, and definite curative clinical efficacy has been achieved. Spleen-strengthening and liver-draining formula (SLF) is a formula formed according to the theory of “One Qi Circulation” of TCM, which originated in the Qing Dynasty (in 1749). SLF is composed of Radix Bupleuri (Chaihu) 9g, Paeoniae Radix Alba (Baishao) 10g, Radix glehniae (Beishashen) 15g, Atractylodis Macrocephalae Rhizoma (Baizhu) 10g, Poria cocos (Fuling) 10g, Citrus Reticulata (Chenpi) 9g, Radix Glycyrrhizae preparate (Gancao) 6g, Sedum sarmentosum (Chuipencao) 15g, Carbonized hawthorn (Shanzhatan) 9g, Salvia miltiorrhiza (Danshen) 15g. In a preliminary clinical observation, SLF showed significant therapeutic effects in patients with NAFLD, including their levels of liver function, blood glucose, and lipids, as well as TCM symptoms.

Recent studies have shown that the imbalance of intestinal flora was closely related to metabolic diseases such as NAFLD, diabetes, and obesity (4). Dysregulation of the intestinal flora is a characteristic of NAFLD, and the signatures of intestinal flora correlate with the severity of the disease by changing bacterial metabolites (4). Furthermore, accumulating evidence suggests that the gut-liver axis is pivotal in NAFLD, especially its the progression to more advanced diseases (5). Consequently, it has been extensively studied for the treatment of this disease by regulating intestinal flora, which has become one of the research focuses in this field.

In this study, we observed the clinical therapeutic result of SLF based on a prospective, randomized, controlled clinical study, and the effect of SLF on intestinal flora of NAFLD was analyzed by 16S rRNA gene sequencing, to further clarify the mechanism of SLF in the treatment of NAFLD.

## Material and methods

### Ethical approval

The study was conducted in conformity with the guidelines set out in the declaration of Helsinki. The study protocol and informed consent were approved by the Institutional Review Board of Shuguang Hospital Affiliated to Shanghai University of Traditional Chinese Medicine (Ethics No. 2020-863-72-01). All the patients who agreed to participate in the trial signed informed consent forms before the trial, and the participants could withdraw from the study at any time freely.

### Study design and participants

This study was designed as a randomized, controlled trial. Participants were recruited from Shuguang Hospital Affiliated to the Shanghai University of Traditional Chinese Medicine from September 2020 to September 2021 who meet the diagnostic of NAFLD. The diagnostic criteria are as follows: (1) No history of alcohol consumption or consumption of less than 30 g of alcohol per day in men (less than 20 g per day in women). (2) Except for certain diseases that can lead to NAFLD including Hepatitis B and C virus, autoimmune liver disease, drug-induced liver disease, genetic metabolic disease, etc. (3) Imaging features of the liver conform with the diagnostic criteria for diffuse fatty liver disease (6).

Eligible patients were between 18 and 70 years old, consistent with the diagnosis of NAFLD; ALT, AST and GGT<5×upper limit of normal (ULN); BMI ≤ 30kg/m^2^; Disease duration≥6 months; Agreed to participate in the trial and sign the informed consent form. Patients excluded if (1) they had liver cirrhosis and other specific diseases which can lead to the fatty liver such as alcoholic liver disease, viral hepatitis, drug-induced liver disease, Wilson’s disease and autoimmune liver disease, etc.; (2) suffered from other serious diseases including malignant tumors, cardiopulmonary diseases, kidney failure and so on; (3) had a history of neurological disease or mental illness; had taken or were required to continuously take lipid-lowering and hepatoprotective drugs within 30 days before enrollment in the trial; (4) were pregnant or nursing, or were planning to get pregnant during the study period; (5) were allergic to the relevant drugs used in the clinic study; (6) or were participating in other clinical trials.

### Interventions description

A screening test had to be conducted for all patients who meet the inclusion criteria during the screening period in clinic. It included the general status of patients, symptoms, and signs associated with the disease, and laboratory detection, including the following: liver function, HOMA-IR, hemorrheologic, FibroTouch, and other examinations. The control group was treated by lifestyle modifications, including diet and exercise. Here, brisk walking was recommended to the patients in this group, and the exercise time had to be ≥150 min per week (7). At the same time, patients had to manage their dietby following a calorie-restricted diet. Patients were to consume, 25kcal/kg/day and reduce their intake of foods and drinks containing fructose (8). On the other hand, the SLF group received SLF formula (use water to decoct twice, filtered liquid after together, simmer to 200mL), twice a day (100mL each time). Besides, diet and exercise which were taken as basic treatment were the same as control group. The intervention was to last for 12 weeks, and we conducted outpatient follow-up visits every 2 weeks during this period.

### Outcomes evaluation

The outcomes included liver function, hepatic fat, blood glucose and lipids, and HOMA-IR. In addition, we recorded the compliance and adverse events of participants, and monitored participants’ health status through blood and urine tests, including kidney function checks and electrocardiogram.

### Stool sample collection

Stool samples were collected from all patients who were recruited with genetic testing sample collectors before and after treatment. Samples were collected only once from HC. All patients were required not to take antibiotics or probiotic preparations in the two weeks preceding the study, and they had to stop eating after 8 PM, before the collection of specimens. They then kept specimens until the next morning in a designated area in the hospital. After collection, specimens were stored at -80°C immediately until further processing. And the stool samples of 11 patients in the SLF group and the control group were randomly selected for subsequent intestinal flora sequencing.

### 16S rRNA gene sequencing analysis

When the raw sequencing data were completed, FastQC was used to check the length and quality of sequencing data control, and errors and low-quality sequences was removed simultaneously. After that, DADA2 (9) was used to generate a variable error model trained on sequencing data for this problem to correct errors and incorporate sequences into amplified sequence variants (ASVs). After quality control and denoising, the feature table and representative sequence were obtained. In this study, QIIME2 (10), which is most widely used in the microbiome, were used to process data as in 16S rRNA gene sequencing analysis. And PICRUSt2 (11) was used to predict functional abundance based on the marker gene sequences. The 16S rRNA gene sequencing was completed with the assistance of Liebing Biotechnology Co., LTD. (Shanghai, China).

### Statistical analysis

All data were statistically analyzed using Graphpad Prism 9.4.0 and R 4.2.1. Measurement data followed a normal distribution, with mean ± standard deviation ( $\bar{x}$ ± s), and don’t follow adopted median (top and bottom quartile). Two independent samples t-test (obeying normal distribution) or Mann-Whitney test were used for comparison among groups; paired t-test (following a normal distribution) or Wilcoxon rank sum test was used for intra-group comparison. Frequency and chi-square test were used for enumeration data. Hierarchical data were expressed by frequency, and comparison between groups was performed through the Mann-Whitney test. The abundance of intestinal flora was expressed by total-sum normalization (TSS). Results were statistically significant when *P*<0.05.

## Results

### Baseline comparison

**Figure 1:**
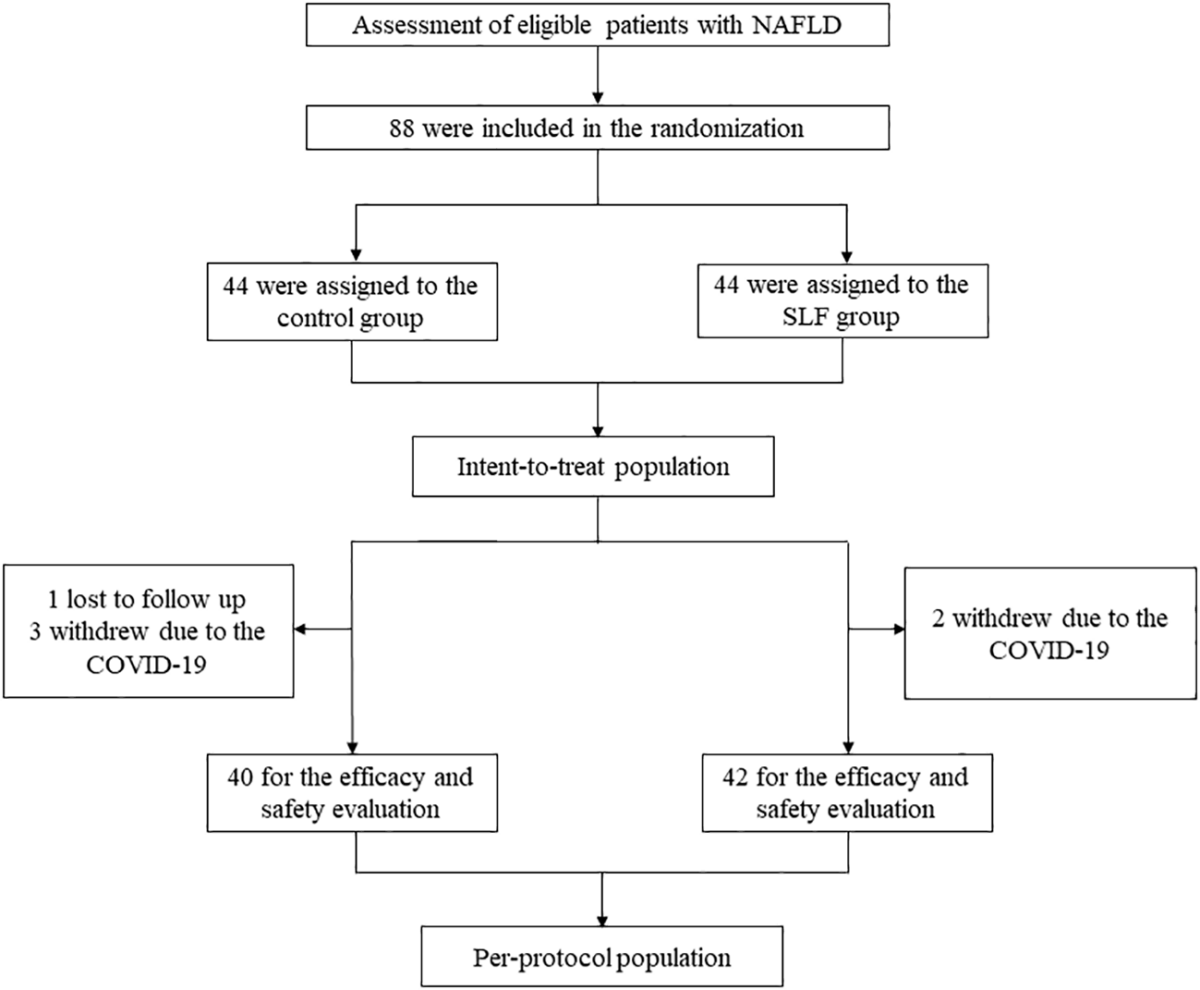
Flow chart of the study. A total of 88 NAFLD patients who were assessed to be eligible were made up of the intent-to-treat population. These patients were randomly divided into the control group and the SLF group, and 1 patient was lost to follow up and 3 patients withdrew due to COVID-19 in the control group, whereas 2 patients in the SLF group. The remaining 82 patients made up the per-protocol population.

After strict inclusion and exclusion criteria, a total of 88 patients were enrolled in this study, with 44 in each group, which made up the intent-to-treat population. In the control group 4 patients were lost to follow up and 2 patients in the treatment group. The final 82 patients (40 in the control group and 42 in the SLF group) were included in the efficacy and safety evaluation, which constituted the per-protocol population (**Figure 1**). There were no statistical differences between the two groups in terms of gender (*P*=0.58), age (*P*=0.09), Classification of fatty liver (*P*=0.92), body mass index (BMI) (*P*=0.54), and onset time (*P*=0.75), which indicated that follow-up comparisons could be conducted (**Table 1**).

**Table 1:** Baseline of the NAFLD in the control group and SLF group.

Index	Group	Control n=40	SLF n=42	*χ2/Z*	*P*
Sex	Male	28	27	0.30	0.58
	Female	12	15		
Age	18-39	22	15	-1.71	0.09
	40-59	15	22		
	≥60	3	5		
Classification of Fatty liver	Mild	13	12	-0.11	0.92
	Moderate	16	21		
	Severe	11	9		
BMI(kg/m^2^)	≤23.9	10	11	-0.61	0.54
	24-27.9	19	23		
	≥28	11	8		
Onset time(Years)	≤1	7	9	-0.32	0.75
	1-5	19	16		
	5-10	11	11		
	>10	3	6		

### SLF had a clinical efficacy on improving the liver function and FibroTouch, as well as relieving symptoms of fatigue

**Figure 2:**
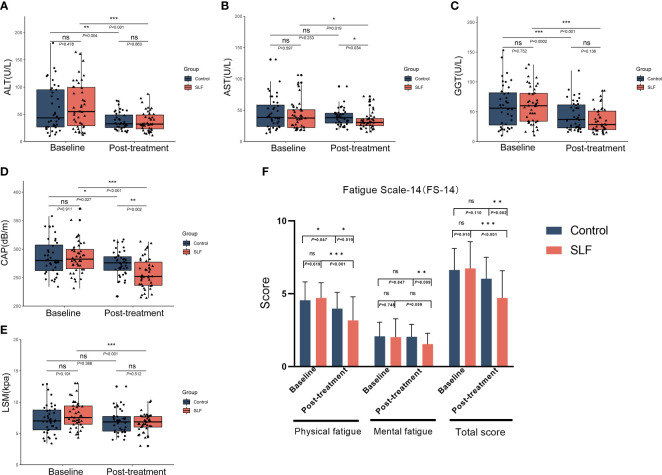
SLF had a clinical efficacy on improving the liver function and FibroTouch, as well as relieving symptoms of fatigue. ALT, AST, GGT, CAP, LSM and FS-14 was compared in the two groups to evaluate the efficacy of SLF in liver function and FibroTouch, as well as the symptoms of fatigue. **(A)** Comparison of ALT in the control group and SLF group. **(B)** Comparison of AST in the control group and SLF group. **(C)** Comparison of GGT in the control group and SLF group. **(D)** Comparison of CAP in the control group and SLF group. **(E)** Comparison of LSM in the control group and SLF group. **(F)** Comparison of FS-14 in the control group and SLF group. ALT, Alanine transaminase; AST, Aspartate aminotransferase; GGT, Gamma glutamyl transpeptidase; CAP, Controlled attenuation parameter; LSM, Liver stiffness measurement; FS-14, Fatigue scale-14. ns, no significance. *P < 0.05, **P < 0.01 and ***P < 0.001.

Liver function was tested for normality in both groups, and ALT, AST, and gamma glutamyl transpeptidase (GGT) did not conform to a normal distribution (*P*<0.05). The differences in ALT, AST, and GGT between the two groups before treatment were not statistically significant and could be compared. According to the results after treatment, differences in the group were performed first, ALT (*P*=0.004) and GGT (*P*<0.001) in the control group were statistically different than before receiving treatment, but not in AST (*P*=0.233). ALT (*P*<0.001), AST (*P*=0.020), and GGT (*P*<0.001) in the SLF group also decreased significantly. Next, when comparing between groups, there was a statistical difference in AST (*P*=0.034), but not in ALT (*P*=0.663) and GGT (*P*=0.136) in the SLF group compared with the control group. (**Figure 2A-C**).

We also compared FibroTouch before and after treatment in both groups. There was no statistical difference in CAP between the two groups pre-treatment (*P*=0.911), but a statistical difference was shown in both groups after treatment when compared to pre-treatment (*P*=0.027 in the control group and *P*<0.001 in the SLF group). However, when the control group and SLF group were compared, there was a statistically significant difference in CAP after treatment (*P*=0.002). Similarly, SLF could improve LSM to a certain extent statistically (*P*=0.001) (**Figure 2D-E**).

In our clinical practice, we found that patients with NAFLD are often accompanied with fatigue symptoms. Therefore, we use the fatigue scale-14 (FS-14) to assess the fatigue symptoms of patients, which includes physical fatigue and mental fatigue (12). We eventually found that SLF improved the physical fatigue (*P*<0.001) as well as the total score (*P*<0.001) of the patients. Although no significant change was found in mental fatigue in the SLF group (*P*=0.059), there was a significant difference compared with the control group after treatment (*P*=0.009) (**Figure 2F**).

### SLF could improve glycolipid metabolism with a good security

**Figure 3:**
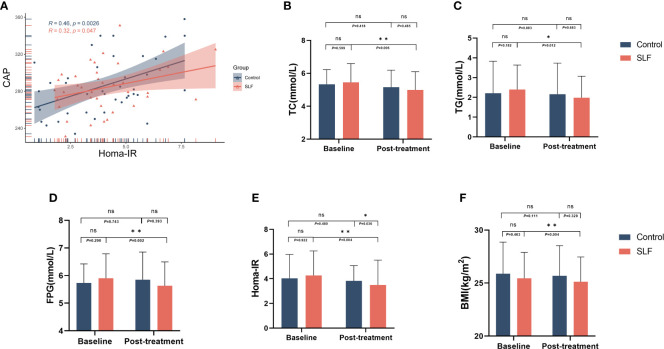
SLF could improve glycolipid metabolism. TC, TG, FPG, HOMA-IR, and BMI were compared to evaluate the efficacy of SLF on metabolism, especially glycolipid metabolism. **(A)** Correlation of HOMA-IR and CAP. **(B)** Comparison of TC in the control group and SLF group. **(C)** Comparison of TG in the control group and SLF group. **(D)** Comparison of FPG in the control group and SLF group. **(E)** Comparison of HOMA-IR in the control group and SLF group. **(F)** Comparison of BMI in the control group and SLF group. TC, Total cholesterol; TG, Triglyceride; FPG, Fasting blood glucose; HOMA-IR, Homeostasis model assessment of insulin resistance; BMI, Body mass index. ns, no significance. *P < 0.05 and **P < 0.01.

In order to investigate the effects of SLF on blood lipids, we conducted a statistical analysis of total cholesterol (TC) and triglyceride (TG) of the two groups. After treatment in the control group, TC (*P*=0.418) and TG (*P*=0.877) were not statistically different than baseline. While in the SLF group, TC (*P*=0.006) and TG (*P*=0.011) were significantly lowered (**Figure 3B, C**), but low-density lipoprotein (LDL) (*P*=0.147), high density lipoprotein (HDL) (*P*=0.320), and free fatty acids (FFA) (*P*=0.060) were not statistically different than before treatment.

IR is closely related to the development of NAFLD, and we compared the correlation between HOMA-IR and CAP and found that they had a positive correlation (r=0.46 in the control group and r=0.32 in the SLF group) (**Figure 3A**). Patients’ fasting plasma glucose (FPG) and fasting insulin (FINS), which were used to calculate HOMA-IR, were measured before and after enrollment in the group. The FPG was not statistically different between the two groups before treatment. When comparing within groups, there was no statistical difference in FPG (*P*=0.753) in the control group compared to baseline, whereas a statistical difference was shown in the SLF group (*P*=0.002) (**Figure 3D**). The patients’ HOMA-IR was calculated by using FPG and FINS, and the ability of SLF to improve IR was evaluated by comparing HOMA-IR between the two groups. Surprisingly, HOMA-IR had improved in the SLF group (*P*= 0.004) (**Figure 3E**). We also compared the body mass index (BMI) of patients in the two groups, and a statistically significant difference was observed in the SLF group (*P*=0.004) (**Figure 3F**).

We compared the safety indicators between the control group and SLF group, and found that the white blood cell (WBC), red blood cell (RBC), platelet (PLT), neutrophil granulocyte (GRA), hemoglobin (Hb), urea nitrogen (BUN), creatinine (Cr) and glomerular filtration rate (GFR) within groups had no statistically significant difference (*P*>0.05), which indicated that the SLF has a good security (**Table 2**).

**Table 2:** Comparison of safety indicators between the control group and SLF group.

Index	Group	Baseline	Post-treatment	*P*
WBC(×10^9^/L)	Control	5.98 ± 1.08	5.86 ± 1.44	0.643
	SLF	6.12 ± 1.23	6.20 ± 1.46	0.792
RBC(×10^12^/L)	Control	4.98 ± 0.48	5.12 ± 0.56	0.202
	SLF	4.99 ± 0.50	5.00 ± 0.67	0.884
PLT(×10^9^/L)	Control	227.68 ± 53.22	247.34 ± 53.05	0.089
	SLF	227.90 ± 50.62	242.93 ± 62.91	0.231
GRA(%)	Control	55.84 ± 8.90	53.62 ± 7.61	0.164
	SLF	55.71 ± 7.12	54.12 ± 6.66	0.246
Hb(g/L)	Control	149.12 ± 15.63	152.67 ± 13.07	0.247
	SLF	152.63 ± 17.08	153.41 ± 16.11	0.820
BUN(mmol/L)	Control	5.04 ± 1.05	5.30 ± 1.23	0.260
	SLF	5.04 ± 1.00	5.48 ± 1.36	0.069
Cr(umol/L)	Control	72.75 ± 15.91	68.78 ± 14.07	0.179
	SLF	71.22 ± 18.41	70.62 ± 13.44	0.840
GFR	Control	102.52 ± 14.00	98.08 ± 14.47	0.081
(ml/(min×1.73m^2^))	SLF	99.60 ± 15.47	98.97 ± 16.55	0.810

### Reduced species diversity and altered intestinal flora abundance were observed in NAFLD

**Figure 4:**
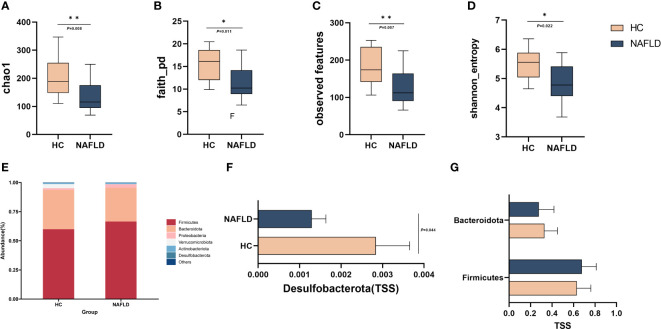
Reduced species diversity and altered intestinal flora abundance were observed in NAFLD. **(A-D)** Comparison of intestinal flora alpha diversity in HC and NAFLD. **(E)**The abundance percentages of HC and NAFLD groups at the phylum level. **(F)** Comparison of the abundance of *Desulfobacterota* in the phylum level. **(G)** Comparison of the abundance of *Firmicutes* and *Bacteroidetes* in the phylum level. TSS, Total-sum normalization. *P < 0.05 and **P < 0.01.

We randomly selected 11 patients, each from the control group and SLF group, and stool specimens were collected from these patients before and after treatment and sent for examination. A total of 53 stool samples (44 NAFLD and 9 HC) were detected utilizing 16S rRNA gene sequencing to analyze the changes of intestinal flora for the mechanism of the potential therapeutic effect of NAFLD by SLF. Alpha diversity was used to observe the diversity of the flora between the HC and NAFLD. We found that there were significant differences in chao1 (*P*=0.0085), Shannon entropy (*P*=0.02), observed features (*P*=0.0065) and faith_pd (*P*=0.009), which indicated reduced species diversity in NAFLD (**Figures 4A-D**). Unfortunately, differences in beta diversity between the HC and NAFLD were not observed (**Figure S1A**).

**Figure 5:**
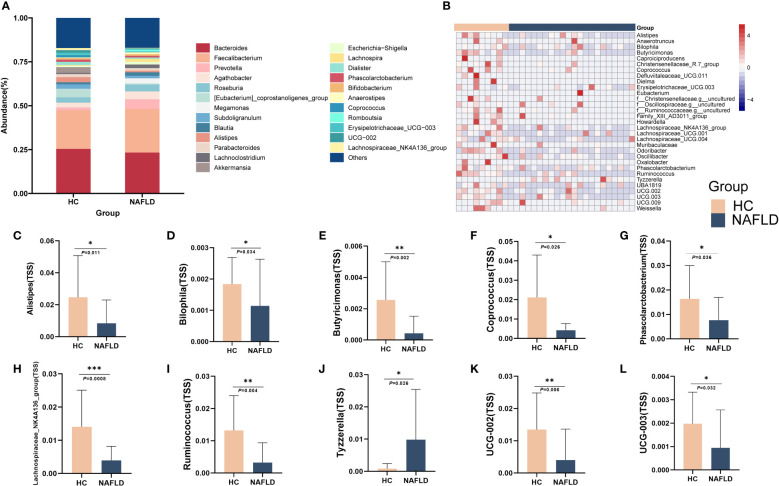
Altered intestinal flora abundance at genus level was observed in NAFLD. **(A)** The abundance percentages of HC and NAFLD groups at the genus level. **(B)** The heatmap of some of the intestinal flora genera with differences between HC and NAFLD. **(C-L)** Comparison of *Alistipes*, *Bilophila*, *Butyricimonas*, *Coprococcus*, *Erysipelotrichaceae_UCG-003*, *Lachnospiraceae NK4A136 group*, *Phascolarctobacterium*, *Ruminococcus*, *Tyzzerell* and *UCG-002* in genus level. TSS, Total-sum normalization. *P < 0.05, **P < 0.01 and ***P < 0.001.

We then compared the abundance percentages of HC and NAFLD groups at the phylum to genus levels, and found that there were varying degrees of alterations in each level (**Figure 4E**, **5A**, **Figure S1B-D**). Differentially abundant taxa were observed in **Figure S2**, which were analyzed by linear discriminant analysis effect size (LEfSe). At the phylum level, *Firmicutes*, *Bacteroidetes*, *Actinobacteriota*, *Desulfobacterota*, and *Proteobacteria* were compared to observe the difference between the two groups. The results showed that *Desulfobacterota* was significantly lower in NAFLD patients when compared with HC (*P*=0.044) (**Figure 4F**). In contrast, there were no significant differences in the other phyla for the time being (**Figure 4G**).

At the genus level, the levels of *Alistipes*, *Bilophila*, *Butyricimonas*, *Coprococcus*, *Erysipelotrichaceae_UCG-003*, *Lachnospiraceae NK4A136* group, *Phascolarctobacterium*, *Ruminococcus* and *UCG-002* were reduced, whereas the abundance of *Tyzzerella* increased (**Figure 5C-L**). The heatmap showed the difference in the genus level between the two groups, indicating that NAFLD may be associated with changes in the abundance of these intestinal flora (**Figure 5B**).

### Intestinal flora correlated with clinical indicators of NAFLD, and SLF might play a role in regulating *Firmicutes* and *Bacteroidetes*

**Figure 6:**
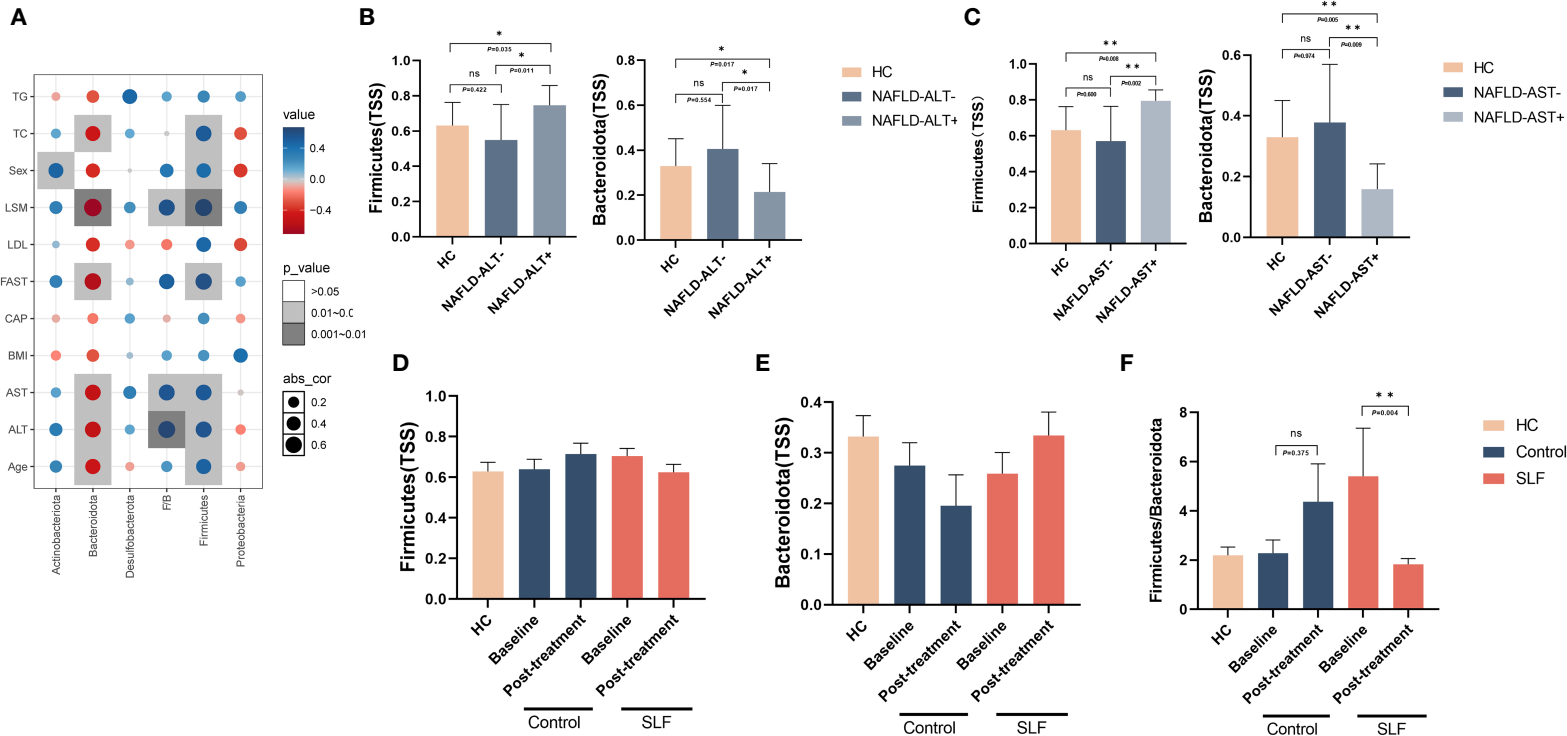
Intestinal flora were correlated with clinical indicators of NAFLD, and SLF might play a role in regulating *Firmicutes* and *Bacteroidetes*. **(A)** Correlation analysis between *Firmicutes*, *Bacteroidetes*, *Actinobacteriota*, *Desulfobacterota*, *Proteobacteria* and F/B with FibroTouch, lipids, glucose, gender, age, liver function and FAST score. **(B)** Comparison of *Firmicutes* and *Bacteroidetes* levels by ALT subgroup. **(C)** Comparison of *Firmicutes* and *Bacteroidetes* levels by AST subgroup. **(D)** Changes in *Firmicutes* levels in the two groups before and after treatment. **(E)** Changes in *Bacteroidetes* levels in the two groups before and after treatment. **(F)** Changes in F/B in the two groups before and after treatment. F/B: The ratio of *Firmicutes* and *Bacteroidetes*. TSS, Total-sum normalization. ns, no significance. *P < 0.05 and **P < 0.01.

To further investigate whether intestinal flora affected the severity of NAFLD, we investigated the correlation between *Firmicutes*, *Bacteroidetes*, *Actinobacteriota*, *Desulfobacterota* and *Proteobacteria* bacteria and FibroTouch, lipids, glucose, gender, age, liver function and Fibroscan-AST (FAST) score(a novel diagnostic signature in NAFLD) (13). Clinical correlation analysis indicated that *Firmicutes* positively correlated with gender, age, ALT, AST, LSM, and FAST score, while the opposite was true for *Bacteroidetes* (**Figure 6A**). When grouped by ALT and AST levels, we found that patients with abnormal ALT or AST had higher level of *Firmicutes* phylum and lower level of *Bacteroidetes* (**Figures 6B, C**), suggesting that the abundance of *Firmicutes* and *Bacteroidetes* may be related to the severity of the disease.

Our clinical trial results showed that SLF ameliorated the condition of NAFLD patients. Whether SLF played a therapeutic role of altering the disturbance of the intestinal flora was still unclear. Consequently, we observed the changes in intestinal flora before and after treatment. Surprisingly, we found that SLF may regulate *Firmicutes* and *Bacteroidetes* levels. Although compared with pre-treatment, *Firmicutes* and *Bacteroidetes* levels in the SLF group were not a statistically significant difference, but they both tended to retrace (**Figures 6D, E**), and the ratio of *Firmicutes* and *Bacteroidetes* was significantly reduced in the SLF group (*P*=0.004) (**Figure 6F**).

### SLF exerted its effect by regulating the disturbance of specific intestinal flora genera

**Figure 7:**
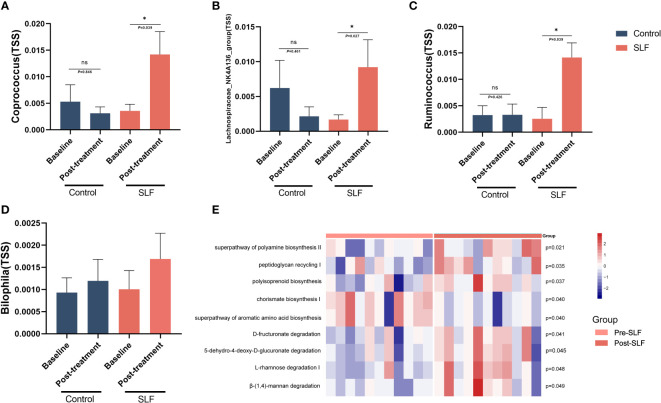
SLF exerted its effect by regulating the disturbance of specific intestinal flora genera. **(A)** Comparison of the level of *Coprococcus* genus in control and SLF group. **(B)** Comparison of the level of *Lachnospiraceae_NK4A136 group* genus in control and SLF group. **(C)** Comparison of the level of *Ruminococcus* genus in control and SLF group. **(D)** Comparison of the level of *Bilophila* genus in control and SLF group. **(E)** The heatmap of differential metabolic pathways of intestinal flora between pre-SLF and post-SLF. TSS: Total-sum normalization. *P < 0.05.

At the genus level, some of the intestinal flora abundances altered. In our study, we found *Coprococcus* (*Lachnospiraceae* family), *Lachnospiraceae_NK4A136 group* (*Lachnospiraceae* family), and *Ruminococcus* (*Ruminococcaceae* family) were significantly higher in the SLF group after treatment, and there was also a tendency for *Bilophila* (*Desulfovibrionaceae* family) to be back-regulated toward HC (**Figures 7A-D**). In addition, at the genus level, SLF could also enhance the level of *Butyricicoccus* and *Blautia*, implying that SLF relieved the symptoms of patients with NAFLD by regulating the disturbance of intestinal flora.

We applied PICRUSt2 to predict the metabolic function of intestinal flora. Metacyc pathway enrichment analysis showed enhanced super pathway of polyamine biosynthesis II (*P*=0.021), peptidoglycan recycling I (*P*=0.035), polyisoprenoid biosynthesis (*P*=0.037), D-fructuronate degradation (*P*=0.041), 4-deoxy-L-threo-hex-4-enopyranuronate degradation (*P*=0.045), L-rhamnose degradation I (*P*=0.048) and β-(1,4)-mannan degradation (*P*=0.049) after treatment, whereas chorismate biosynthesis I (*P*=0.040) and super pathway of aromatic amino acid biosynthesis (*P*=0.040) decreased (**Figure 7E**).

## Discussion

NAFLD is often accompanied with disorders of glucolipid metabolism (14–16), which may be the most common initial predisposing factors for the development of NAFLD (16). IR, an important feature of NAFLD, is present in all stages of NAFLD development. Circulating insulin and glucose promotes *de novo* lipogenesis (DNL) through activation of sterol regulating element binding protein 1c (SREBP1c) and the carbohydrate response element binding protein (ChREBP) (17, 18). It can promote the prevention and treatment of NAFLD through regulating hepatocyte glucolipid metabolism (19). In the early stages of NAFLD, restoration of metabolic disorders through herbal discriminatory treatment may inhibit the development of NAFLD. TCM, as a major part of comprehensive treatment, plays a key role in the treatment of NAFLD. SLF is a formula formed based on the traditional theory of “One Qi Circulation”, which has the function of strengthening the spleen, resolving dampness and draining the liver to relieve depression. In our study, SLF has a definitive efficacy in improving liver function and glucolipid metabolism of NAFLD, as well as relieving the symptoms of fatigue, which may become an adjuvant therapy in the clinical treatment of NAFLD patients.

The study of intestinal flora has become a hot spot in the study of metabolic diseases and microorganisms has grown rapidly in the past few decades. The intestinal flora participates in the absorption and metabolism of nutrients (including the metabolism of carbohydrates, lipids, and amino acids) in the human body, which plays a strong part in maintaining fitness (20). It has been proved that intestinal flora could influence the microbiota-gut-liver axis to regulate intestinal metabolism (20). Increasing evidence indicates that there are multiple links between intestinal flora and hepatic steatosis: (1) Appetite signal of the host is influenced by it; (2) It can also increase energy extraction from the intestine; (3) Changes in the metabolism of bile acids, which affect the fat and lipid vitamins obtained in the intestine; (4) Modulation of choline metabolism; (5) Promotion of inflammation in host organisms; (6) Bowel bacterial overgrowth and increased intestinal permeability will contribute to bacteria translate into the systemic circulation and endotoxemia (21–24). Furthermore, study found that alterations in the dominant intestinal flora and the abundance and diversity of microbial composition decreased in NAFLD patients (25). Compared with healthy people, the levels of *Firmicutes* and *Proteobacteria* in the gut of NAFLD patients were significantly increased (25, 26).In addition, research shows that intestinal flora plays a vital role in disorders of glucolipid metabolism. The individuals with glucolipid metabolic disorders are always accompanied by intestinal flora disorders and decreased diversity compared to normal individuals (27). In recent years, it has been found that the disorder of intestinal flora may be one of the main causes of the disorder of glucolipid metabolic (28). Therefore, it is considered as a potential therapeutic target for the prevention and treatment of NAFLD and disorders of glycolipid metabolism by regulating the intestinal flora.

As a consequence, we speculate there is a link between abnormal intestinal flora and NAFLD. With the increased understanding of the “gut-liver axis” and the popularity of fecal amplicon sequencing, the gut microbiome seems to occupy an undisputed position in the pathogenesis of NAFLD. Similar to our study, the diversity and abundance of intestinal flora in NAFLD have been shown to change to varying degrees in both animal and human studies (29–31). Therefore, it may be helpful for the prevention and treatment of NAFLD by means of adjusting the intestinal microecology, restoring the normal interaction between “intestinal flora and host”, alleviating IR, and promoting glucose and lipid metabolism.

The clinical investigations and animal experiments have showed that TCM could reverse the dysregulation of intestinal flora and maintain the balance of intestinal micro-ecological system (32).This clinical efficacy may be the result of SLF regulation of intestinal flora and there are some pieces of evidence that herbs in SLF may regulate the intestinal flora. Studies have shown that radix bupleuri could increase intestinal flora diversity and decrease the level of *Prevotella* and *Ochrobactrum (* 33). Aqueous extract of Paeoniae Radix Alba could regulate the intestinal mucosal barrier and increase the level of *norank_f_Muribaculaceae*, *Lactobacillus*, *Akkermansia*, etc. (34). Poria cocos oligosaccharides could improve disorders of glucolipid metabolism by regulating intestinal flora (35). Salvia miltiorrhiza Polysaccharide combined with probiotics can improve insulin resistance and NAFLD *via* modulating intestinal flora (36). In addition, Atractylodes macrocephala Koidz and Citrus also have the function of regulating intestinal flora (37, 38). Glycyrrhizic acid could stabilize intestinal flora in chronic liver injury through increased probiotics and decreased pernicious bacteria (39). Procyanidins from hawthorn supplementation significantly relieved lipid accumulation in the serum and liver, and protected the structure of liver in lipid metabolism disorder (LMD) rats. Procyanidins from hawthorn (HPC) especially increased the abundances of *Akkermansia*, *Bacteroides* and *Adlercreutzia*, and decreased *Lactobacillus*, *Bifidobacterium*, *Blautia*, *Lachnospiraceae* and *Subdoligranulum*, which could also regulated the structure of intestinal flora (40).

We collected the stool samples of patients who were diagnosed with NAFLD and HC to examine the composition and abundance of the intestinal flora *via* 16S rRNA high-throughput gene sequencing technology to further validate our view based on the hypothesis that NAFLD is related to the abnormal intestinal flora. Intestinal flora may be affected by different living habit; hence, we ensured the same lifestyle intervention of SLF and control groups. Although there were no differences observed in *Proteobacteria* and *Fusobacteria* phyla, the phylum *Desulfobacterota* was significantly lower in NAFLD. As the two major phyla of the intestinal flora, *Firmicutes* and *Bacteroidetes* may be altered in patients with NAFLD and affect the development of NAFLD. A trial comparing the human intestinal flora of NAFLD (n=25) and HC (n=22) found that NAFLD patients had a lower abundance of the *Bacteroidetes (* 31). In other experiments, the ratio of *Firmicutes* and *Bacteroidetes* was significantly higher in NAFLD patients (41, 42). Although opposite results have also been found (43, 44), our study identified clinically relevant alterations in the *Firmicutes* and *Bacteroidetes* phyla, which may play a critical role in the development of NAFLD. In particular, we found a positive correlation between the Firmicutes phylum and the FAST score (13), a potential signature for non-invasive diagnosis of NASH, while the opposite for the *Bacteroidetes* phylum.

In our study, significant changes occurred in *Coprococcus*, *Lachnospiraceae _NK4A136* group and *Ruminococcus* after SLF treatment. *Coprococcus* is capable of actively fermenting carbohydrates and is one of the important producers of butyric acid. *Lachnospiraceae_NK4A136* group belongs to the family *Lachnospiraceae* and is a potentially beneficial bacterium associated with obesity. It is also related to the production of butyric acid (45, 46). *Ruminococcus* play a crucial role in metabolism. It has been shown that the level of *Ruminococcus* is reduced in patients with NAFLD (41), which is similar to our results. Whereas SLF could call back the level of *Ruminococcus*. These results suggest that SLF may treat NAFLD by modulating certain specific enterobacterial genera.

Nonetheless, our study has some shortcomings. The treatment course of NAFLD patients is relatively short, and the sample size is not enough, which leads to not obvious differences and incomplete recovery of the disorder flora after SLF treatment. In subsequent studies, we will expand the sample size to further confirm the effect of SLF on intestinal flora in NAFLD patients. In addition, we only observed the effect of SLF on different classification levels of intestinal flora in patients. In future research, we will further study the potential mechanisms of different strains of SLF.

## Conclusion

Our study suggested that NAFLD had relations with disturbances in the intestinal flora, which manifested as the levels of *Tyzzerella* increased and *Alistipes*, *Bilophila*, *Butyricimonas*, *Coprococcus*, *Erysipelotrichaceae UCG-003*, *Lachnospiraceae NK4A136* group, *Phascolarctobacterium*, *Ruminococcus* and *UCG-002* decreased. SLF could improve liver function and glucolipid metabolism in patients who were diagnosed with NAFLD and lower liver fat content to some extent. The effect of SLF may be carried out by regulating the disturbance of intestinal flora, especially *Coprococcus*, *Lachnospiraceae_NK4A136* group, and *Ruminococcus* genus.

## Data availability statement

The data presented in the study are deposited in the NCBI repository, accession number PRJNA921570.

## Ethics statement

The studies involving human participants were reviewed and approved by the Institutional Review Board of Shuguang Hospital Affiliated to Shanghai University of Traditional Chinese Medicine (Ethics No. 2020-863-72-01). The patients/participants provided their written informed consent to participate in this study.

## Author contributions

DH: Project management, Methodology, Writing manuscript. LL: Conceptualization, Investigation, Methodology. NA:Conceptualization, Revision. JS, YH and WX: Visualization. CW,DX and YJ: Revision. YB: Supervision, Methodology. MS: Funding acquisition, Writing – review and editing. All authors contributed to the article and approved the submitted version.

## Supplementary material

Supplementary Figure 1
**(A)** Comparison of intestinal flora beta diversity in HC and NAFLD, including unweighted and weighted Unifrac PCoA. **(B)** The abundance percentages of HC and NAFLD groups at the class level. **(C)** The abundance percentages of HC and NAFLD groups at the order level. **(D)** The abundance percentages of HC and NAFLD groups at the family level.

Supplementary Figure 2 Differentially abundant taxa between HC and NAFLD samples analyzed by linear discriminant analysis effect size (LEfSe).
