# Supplementary figures and images for: The spleen-strengthening and liver-draining herbal formula treatment of non-alcoholic fatty liver disease by regulation of intestinal flora in clinical trial
Source: Front Endocrinol (Lausanne). 2023 Jan 19;13:1107071. doi: 10.3389/fendo.2022.1107071 (PMC9892935; doi:10.3389/fendo.2022.1107071)

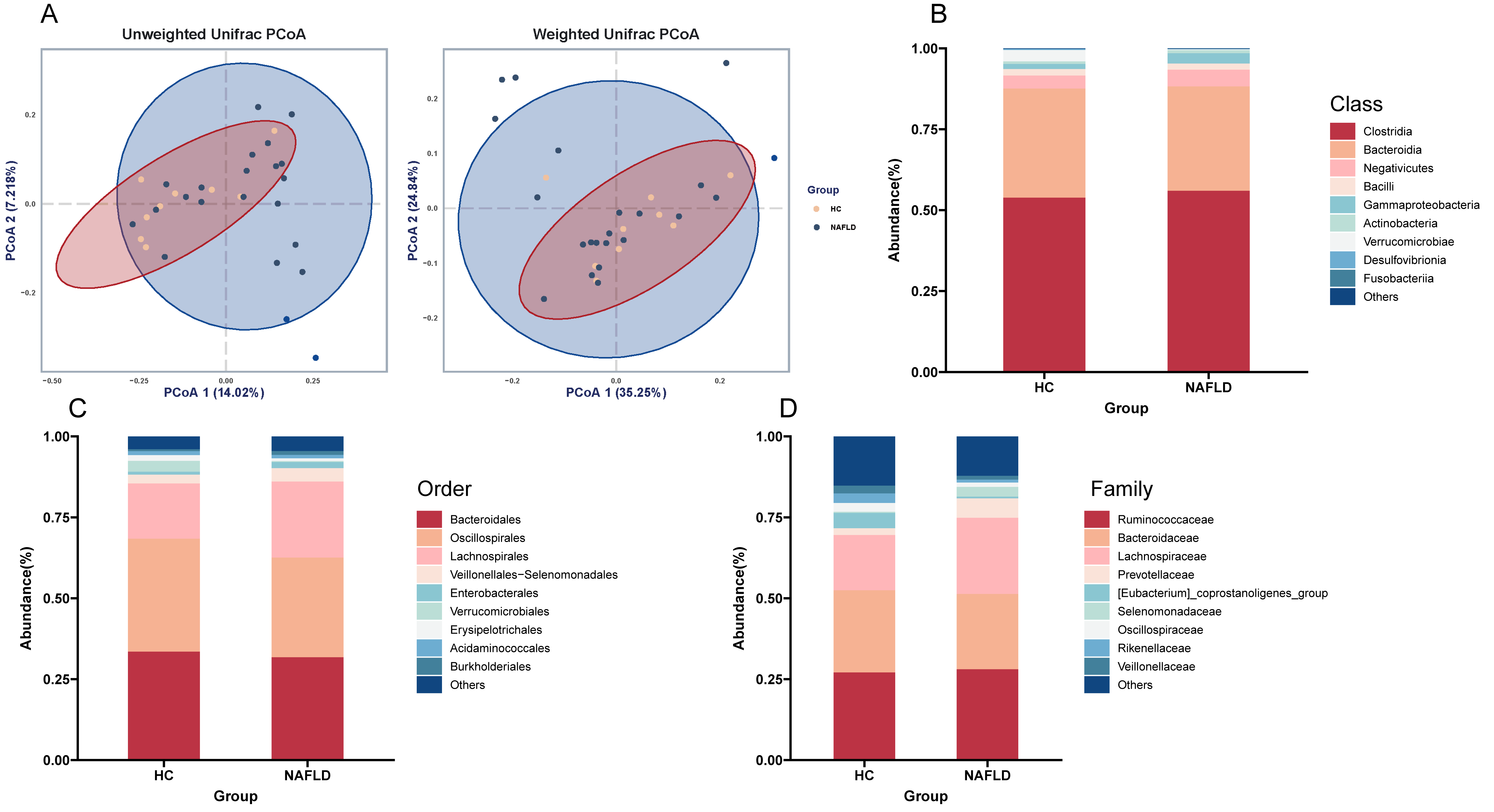

Supplement: Supplementary Figure 1 — (A) Comparison of intestinal flora beta diversity in HC and NAFLD, including unweighted and weighted Unifrac PCoA. (B) The abundance percentages of HC and NAFLD groups at the class level. (C) The abundance percentages of HC and NAFLD groups at the order level. (D) The abundance percentages of HC and NAFLD groups at the family level. [file Image_1.tif]

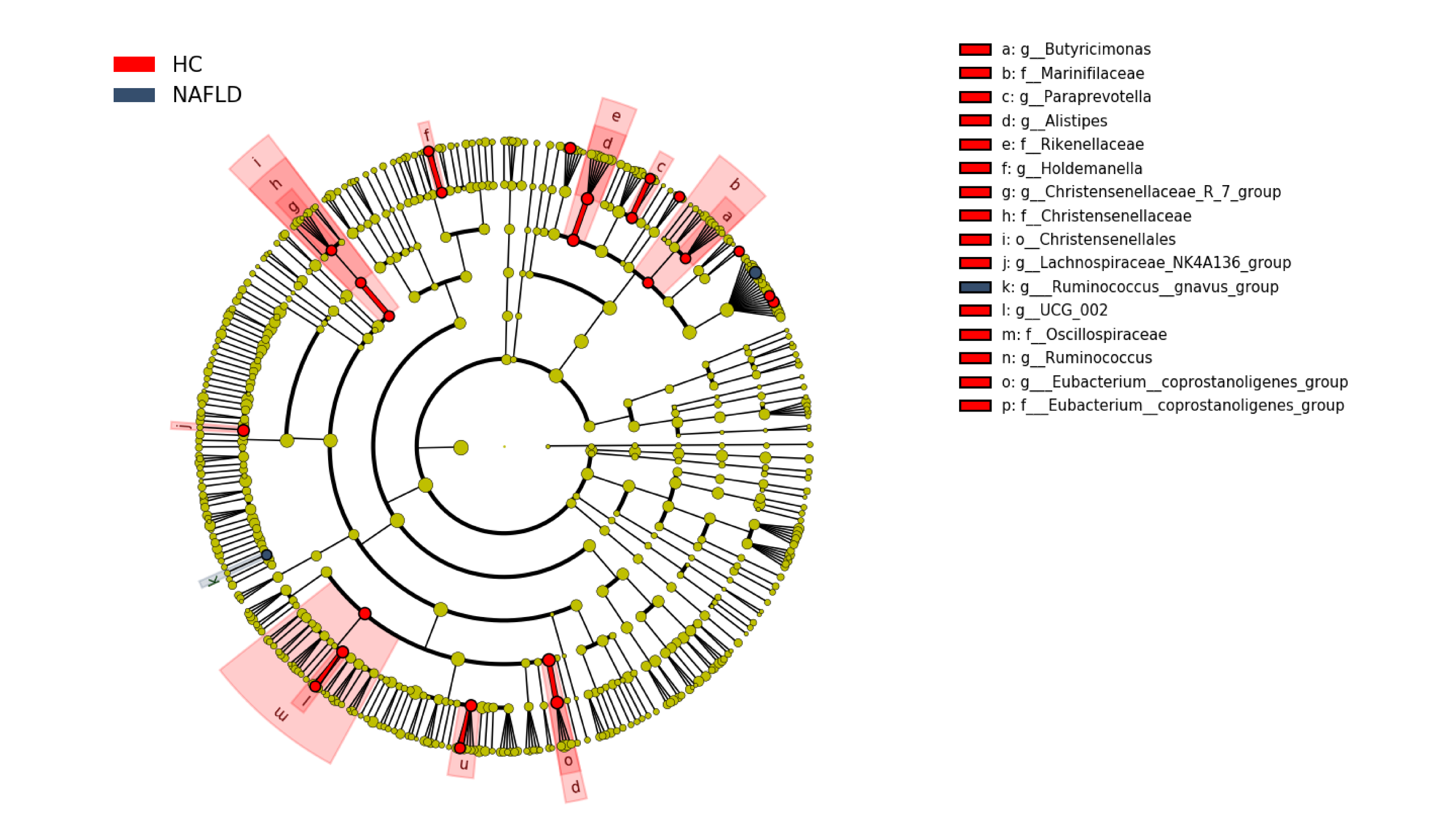

Supplement: Supplementary Figure 2 — Differentially abundant taxa between HC and NAFLD samples analyzed by linear discriminant analysis effect size (LEfSe). [file Image_2.tif]
